# Association between red blood cell distribution width and psoriasis among the US adults

**DOI:** 10.3389/fmed.2023.1290514

**Published:** 2023-12-21

**Authors:** Yunqi Zhang, Zheng Lv, Peng Peng, Tie Zhao

**Affiliations:** ^1^Department of Pharmacy, Guangzhou Institute of Dermatology, Guangzhou, China; ^2^The First Affiliated Hospital, Guangzhou University of Chinese Medicine, Guangzhou, China

**Keywords:** red blood cell distribution width, psoriasis, NHANES, cross-sectional study, female

## Abstract

**Introduction:**

The association between red blood cell distribution width (RDW) and psoriasis among the US adults is still unknown. We aimed to assess whether RDW is associated with psoriasis in the US adults.

**Method:**

We conducted a cross-sectional study consisting of 14,089 participants from National Health and Nutrition Examination Survey (NHANES) 2009–2014. Psoriasis status were assessed by self-reported questionnaire. We evaluated the association between RDW and risk of psoriasis using multivariate regression models. Subgroup and interaction analysis were performed.

**Results:**

The higher RDW level was associated with an increased risk of psoriasis (OR = 1.10 [95% CI, 1.01, 1.19]; *p* = 0.025) after adjusting for confounders in female. However, there is no significant association between RDW and risk of psoriasis among male (OR = 0.99 [95% CI, 0.87, 1.15]; *p* = 0.992). Subgroup and interaction analysis found that the strongest positive association mainly exists in female participants with BMD greater than 29.9 kg/m^2^ (OR = 1.20 [95% CI, 1.09, 1.32], Pint = 0.004).

**Discussion:**

In conclusion, we found that increased RDW levels were associated with an increased risk of psoriasis in females, which could provide clinicians with auxiliary data for the early diagnosis of psoriasis.

## Introduction

Psoriasis is a chronic autoimmune skin disease believed to be caused by a combination of genetic and environmental factors (1). In the United States, approximately 7.5 million adults have psoriasis (2), with variations in prevalence among different age and ethnic groups. Typical symptoms of psoriasis include red, thick patches covered with silvery scales, dry and intensely itchy skin, as well as cracks, pain, and occasional bleeding (3). Currently, psoriasis remains a not curable disease, but symptoms can be relieved through medication and phototherapy (4, 5). Infections, stress, injury, and certain medications may trigger or exacerbate psoriasis (6, 7). Overall, understanding its causes and risk factors is essential for prevention and management of this disease.

Red Cell Distribution Width (RDW) is a parameter that reflects the proportion of red blood cells of different sizes in the blood and is commonly used in clinical testing and diagnosis. The normal range of RDW is usually between 11.5 and 14.5%, an increased value of RDW indicate an uneven distribution of red blood cell size in various diseases, such as anemia, malnutrition, chronic diseases, infections, and bleeding (8). In recent years, many studies reported that high RDW values are associated with an increased risk of malignant tumors, cardiovascular diseases, and pulmonary diseases (9–11). Moreover, it has been proven to be closely related with inflammatory or autoimmune conditions such as rheumatoid arthritis and systematic lupus erythematosus (12, 13). Meanwhile, chronic inflammation has been reported as leading to the development of psoriasis. In this regard, there is ongoing research involve in detecting of the predictive value of RDW for diagnosing psoriasis. Several studies reported that RDW values in psoriasis patients were uncovered higher than healthy matching (14–16). However, another study have detected no association of RDW value with psoriasis (17). In addition, there is limited data on the association between RDW and psoriasis among the representative and large sample of US population.

Therefore, the aim of this study is to analyze the association between RDW levels and psoriasis in US adults, using data from the nationally representative National Health and Nutrition Examination Survey (NHANES) database (2009–2014). We also aim to provide clinicians with auxiliary data for the early diagnosis of psoriasis.

## Materials and methods

### Study population

**Figure 1:**
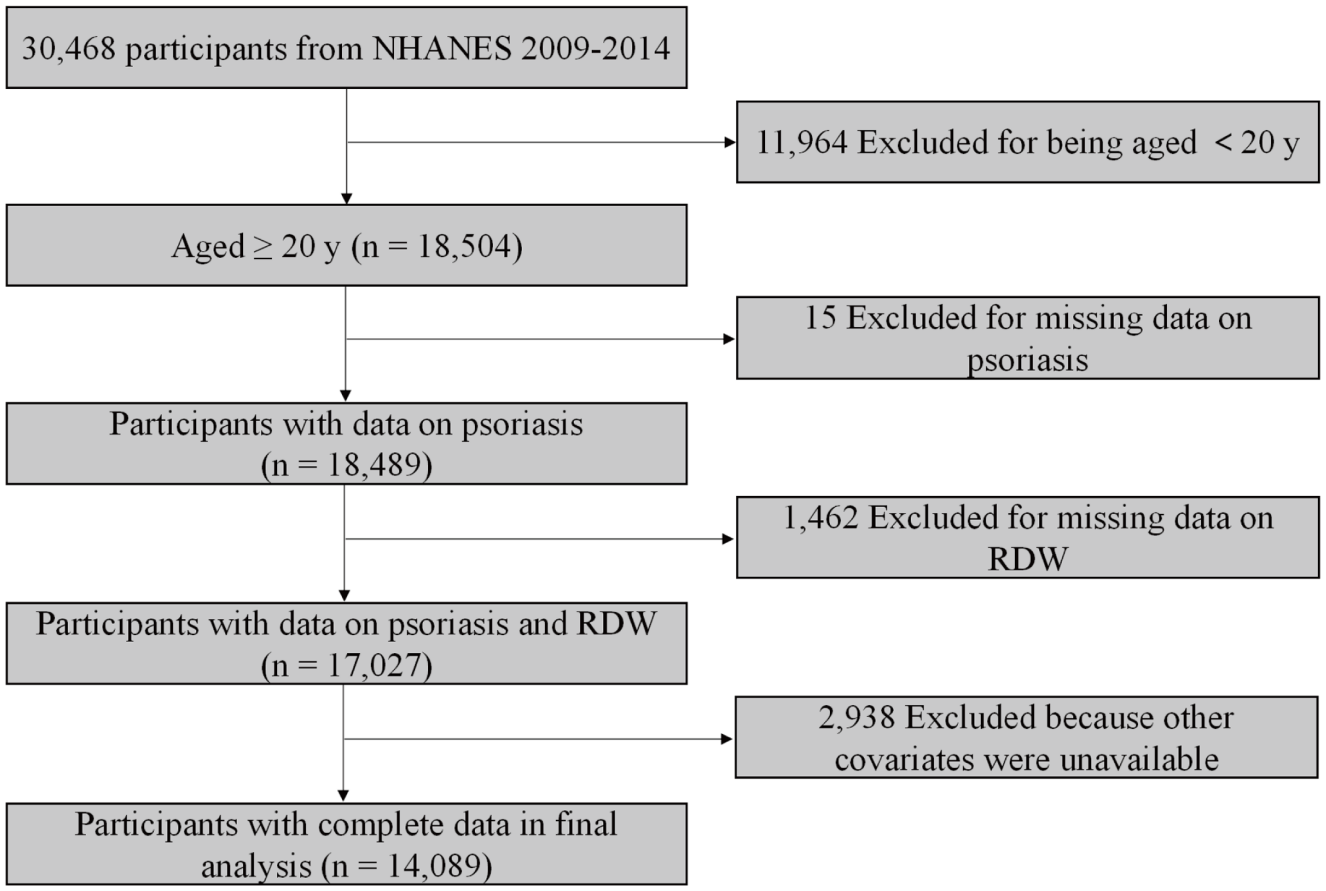
Flowchart of sample selection.

The NHANES database is an ongoing national survey focusing on health and health-related behaviour of the US population. The NHANES database is available publicly at www.cdc.gov/nchs/nhanes. We included data from three 2-year NHANES cycles (2009–2014). In total, 18,504 participants aged greater than 20 years were included. We excluded participants without data on psoriasis (*n* = 15). After further excluding individuals with missing value for RDW (*n* = 1,462) and other covariates (*n* = 2,938), 14,089 participants were included for final analysis (Figure 1).

### Assessment of psoriasis and RDW

Psoriasis was defined as an affirmative response to the question, “Have you ever been told by a health care provider that you had psoriasis?” Whole blood samples were collected from all participants, and RBCs were measured using the Beckman automated Coulter method of counting and sizing (Coulter Counter; Coulter Electronics, Luton, United Kingdom) 40. Mean Corpuscular Volume (MCV) was calculated as the average volume of individual erythrocytes derived from the histogram of RBC. Finally, the RDW (%) was calculated as the SD of MCV divided by the mean of MCV, multiplied by 100.

### Assessment of covariates

Covariates included age, race/ethnicity (including Non-Hispanic white, Non-Hispanic black, Mexicane American, Other Hispanic, and Other ethnicity), body mass index (BMI), physical activity, alcohol drinking status, smoking status, diabetes, congestive heart failure, and stroke. To assess physical activity, weekly metabolic equivalent (MET) minute aggregated scores were calculated for each participant (18). According to the MET-minutes, participants were categorized into inactive (<600 MET-minute/week), moderately active (600–3,000 MET-minute/week), and highly active (> 3000MET-minute/week). Significant alcohol use was determined by the question, “In any 1 year, have you had at least 12 drinks of any type of alcoholic beverage?” Participants who answered “yes” were defined as alcohol drinkers. Smoking history was determined if the patient responded “yes”to ever smoking at least 100 cigarettes. Covariates relating to medical history such as a history of congestive heart failure, stroke, and diabetes mellitus were gathered from specific, individual questions asking participants whether a physician had ever told them that they had the condition of interest. Information on each variable and acquisition process are publicly available at www.cdc.gov/nchs/nhanes.

### Statistical analysis

Data were presented as mean ± standard deviation for continuous variables and as percentages for categorical variables. We grouped participants into quartiles according to hemoglobin levels. We used the ANOVA tests for continuous variables with a normal distribution and Kruskal-Wallis test for continuous variables without a normal distribution. The chi-square tests were performed for categorical variables to assess the characteristics of the participants. Multivariate logistic regression analyses were used to evaluate the relationship between RDW and psoriasis risk with odds ratio (OR) and corresponding 95% confidence interval (CI). We contrasted three models as follows: model 1, no covariate was adjusted; model 2, adjusted for age, and race/ethnicity; model 3, additionally adjusted for BMI, physical activity, alcohol drinking status, smoking status, diabetes, congestive heart failure, and stroke.

In addition, subgroup analyses were also conducted stratified by different age, BMI, race/ethnicity, alcohol drinking status, smoking status, diabetes, congestive heart failure, stroke, and physical activity. A two-sided *p*-value <0.05 was considered statistically significant. Statistical analyses were conducted using the EmpowerStats (http://www.empowerstats.com, X&Y Solutions, Inc., Boston, MA) and statistical software packages R (http://www.R-project.org, The R Foundation).

## Results

### Characteristics of participants

Table 1 presented the characteristics of participant with non-psoriasis and psoriasis. Among males, 189 (2.72%) participants were diagnosed with psoriasis. Participants with psoriasis tend to be older and non-Hispanic White individuals, to have higher mean BMI, and more commonly reported a history of diabetes and smoking. In contrast, 209 (2.93%) participants had psoriasis in females. Participants with psoriasis tend to be older and non-Hispanic White individuals, to have higher mean RDW and BMI, and more commonly reported a history of congestive heart failure and smoking.

**Table 1:** Baseline characteristics of the study subjects according to psoriasis status.

	Male (*n* = 6,945)			Female (*n* = 7,144)		
	No psoriasis (*n* = 6,756)	Psoriasis (*n* = 189)	*p*-value	No psoriasis (*n* = 6,935)	Psoriasis (*n* = 209)	*p*-value
Age (y)	48.75 ± 17.75	52.19 ± 16.27	0.007	49.15 ± 17.69	51.58 ± 17.17	0.049
Race (%)			<0.001			<0.001
Non- Hispanic White	2,976 (44.05%)	113 (59.79%)		3,049 (43.97%)	127 (60.77%)	
Non- Hispanic Black	1,427 (21.12%)	16 (8.47%)		1,441 (20.78%)	24 (11.48%)	
Mexican American	971 (14.37%)	19 (10.05%)		963 (13.89%)	14 (6.70%)	
Other Hispanic	610 (9.03%)	18 (9.52%)		729 (10.51%)	23 (11.00%)	
Other ethnicity	772 (11.43%)	23 (12.17%)		753 (10.86%)	21 (10.05%)	
Alcohol (%)			0.296			0.508
Yes	5,710 (84.52%)	165 (87.30%)		4,290 (61.86%)	134 (64.11%)	
No	1,046 (15.48%)	24 (12.70%)		2,645 (38.14%)	75 (35.89%)	
Diabetes (%)			0.011			0.067
Yes	862 (12.76%)	36 (19.05%)		865 (12.47%)	35 (16.75%)	
No	5,894 (87.24%)	153 (80.95%)		6,070 (87.53%)	174 (83.25%)	
Congestive heart failure (%)			0.110			<0.001
Yes	216 (3.20%)	10 (5.29%)		192 (2.77%)	15 (7.18%)	
No	6,540 (96.80%)	179 (94.71%)		6,743 (97.23%)	194 (92.82%)	
Stroke (%)			0.296			0.652
Yes	231 (3.42%)	8 (4.23%)		192 (2.77%)	15 (7.18%)	
No	6,525 (96.58%)	181 (95.77%)		6,696 (96.55%)	203 (97.13%)	
Smoking status (%)			0.002			0.001
Non-smoker	3,208 (47.48%)	68 (35.98%)		4,450 (64.17%)	111 (53.11%)	
Smoker	5,710 (84.52%)	165 (87.30%)		2,485 (35.83%)	98 (46.89%)	
Physical activity			0.735			0.218
MET minutes <600	3,588 (53.11%)	103 (54.50%)		5,005 (72.17%)	158 (75.60%)	
MET minutes 600–3,000	2,557 (37.85%)	72 (38.10%)		1745 (25.16%)	43 (20.57%)	
MET minutes >600	611 (9.04%)	14 (7.41%)		185 (2.67%)	8 (3.83%)	
RDW	13.10 ± 1.13	13.11 ± 1.16	0.894	13.37 ± 1.53	13.59 ± 1.96	0.043
Body mass index (kg/m^2^)	28.45 ± 6.23	29.34 ± 5.56	0.020	29.41 ± 7.87	30.70 ± 7.86	0.018

### Association between RDW and psoriasis risk

Table 2 showed the association between RDW and psoriasis risk in males group. In the unadjusted model, no significant association between RDW and psoriasis risk was observed (OR = 1.01 [95% CI, 0.89, 1.15]; *p* = 0.843). After adjusting for confounders, RDW was also not significantly associated with psoriasis risk in model 2 (OR = 1.02 [95% CI, 0.89, 1.17]; *p* = 0.737) or model 3 (OR = 0.99 [95% CI, 0.87, 1.15]; *p* = 0.992). Table 3 presented the association between RDW and psoriasis risk in females group. In the unadjusted model, increased RDW levels was significantly correlated with higher psoriasis risk (OR = 1.08 [95% CI, 1.01, 1.16]; *p* = 0.043). After adjusting for age and race/ethnicity, RDW was positively associated with higher risk of psoriasis (OR = 1.12 [95% CI, 1.04, 1.20]; *p* = 0.003) in Model 2. After fully adjusting for confounders, RDW was still positively associated with higher risk of psoriasis (OR = 1.10 [95% CI, 1.01, 1.19]; *p* = 0.025) in Model 3.

**Table 2:** Association of RDW with risk of sarcopenia.

	Male	
	OR (95% CI)	*p* value
Model 1	1.01 (0.89, 1.15)	0.843
Model 2	1.02 (0.89, 1.17)	0.737
Model 3	0.99 (0.87, 1.15)	0.992

**Table 3:** Association of RDW with risk of sarcopenia.

	Female	
	OR (95% CI)	*p* value
Model 1	1.08 (1.01, 1.16)	0.043
Model 2	1.12 (1.04, 1.20)	0.003
Model 3	1.10 (1.01, 1.19)	0.025

**Figure 2:**
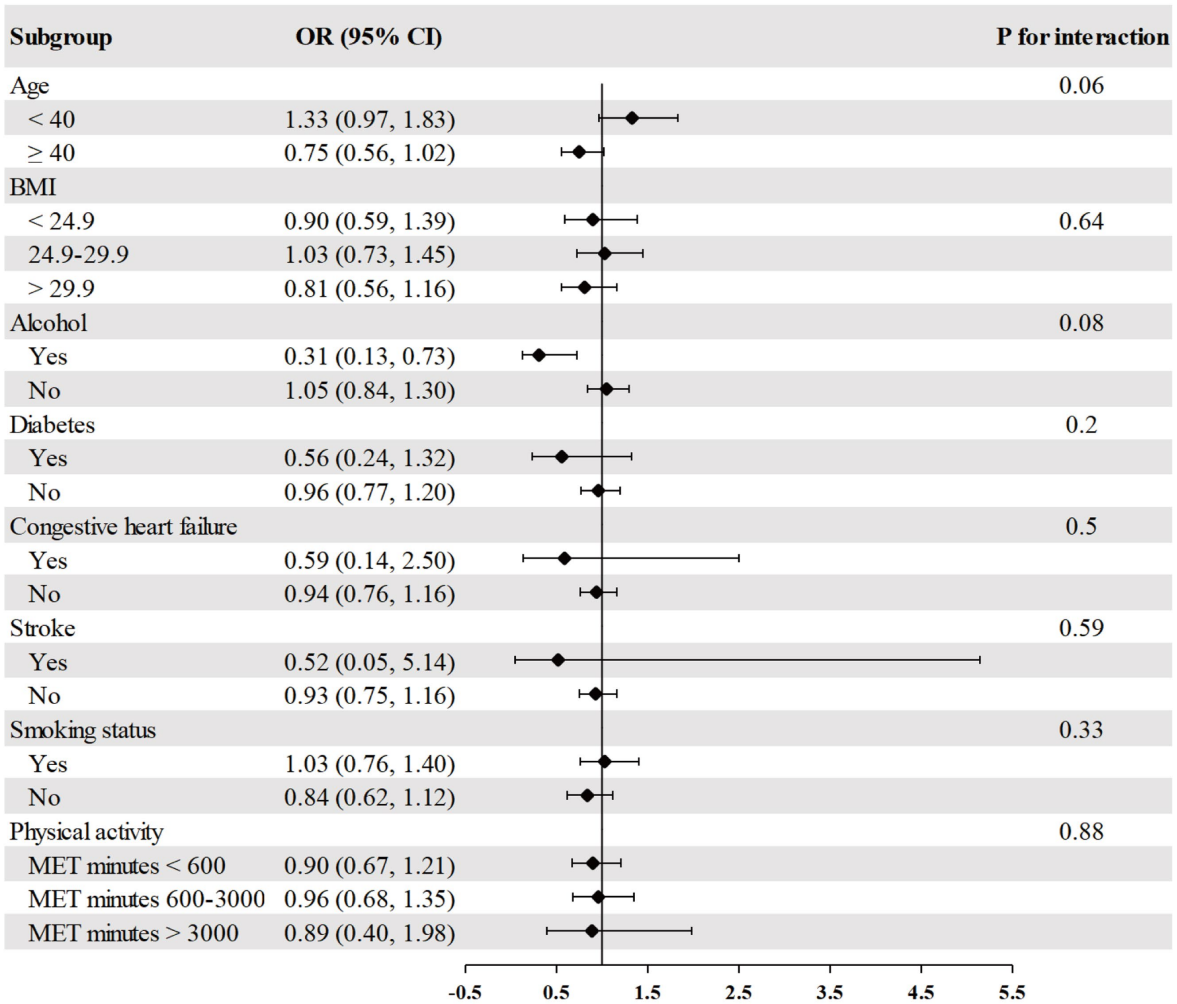
Association between RDW and psoriasis risk in different subgroups among males. Age, race/ethnicity, BMI, physical activity, alcohol drinking status, smoking status, diabetes, congestive heart failure, and stroke were adjusted (the stratified variable was omitted from the model).

**Figure 3:**
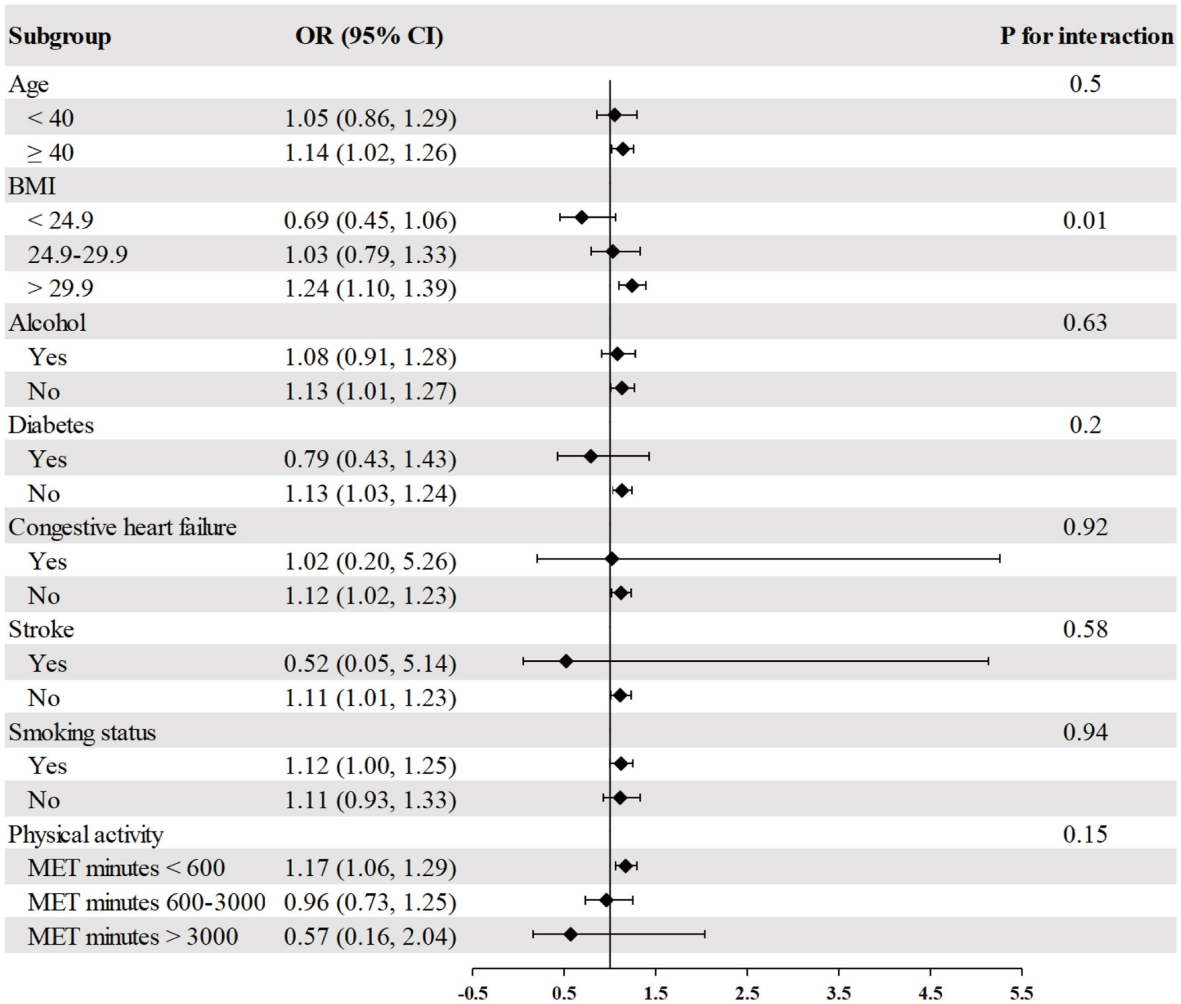
Association between RDW and psoriasis risk in different subgroups among females. Age, race/ethnicity, BMI, physical activity, alcohol drinking status, smoking status, diabetes, congestive heart failure, and stroke were adjusted (the stratified variable was omitted from the model).

The association between RDW and psoriasis risk was not observed in all different subgroups among males, including age, race, BMI, alcohol consumption, diabetes, congestive heart failure, stroke, smoking, and physical activity (Figure 2). We found a significant interaction between RDW and BMI with the risk of psoriasis (*p* = 0.004 for interaction) in females (Figure 3). The association between RDW and higher risk of psoriasis (OR = 1.20 [95% CI, 1.09, 1.32]) was stronger in populations of female with BMI greater than 29.9 kg/m^2^.

## Discussion

In the present study, we used the data from population-based national survey to evaluate the association between RDW and psoriasis risk in US adults. We observed a positive association between RDW and psoriasis risk in females after fully adjusting for covariates. No association between RDW and psoriasis risk was found in males. Subgroup analysis indicated that the association between RDW and psoriasis risk was more pronounced in female with BMI greater than 29.9 kg/m^2^.

Psoriasis is an inflammatory skin disease associated with many other conditions (19). The most obvious complication is psoriatic arthritis, which can be observed in 10–40% of psoriasis patients and usually occurs 10 years after the onset of psoriasis (20, 21). Psoriasis and psoriatic arthritis share some common pathogenic mechanisms and immunological features. Other diseases are more common in psoriasis patients than in the general population, including chronic obstructive pulmonary disease (22), asthma (23), adverse cardiovascular events (MACE) (24), chronic kidney disease (25), and inflammatory bowel disease (IBD) (26). Several retrospective studies reported that RDW can predict the prognosis of chronic obstructive pulmonary disease (27, 28). Conic et al. found that RDW may be a cost-effective clinical tests to identify psoriasis and psoriatic arthritis patients at increased risk of MACE (29). Solak et al. measured RDW value in 367 patients with chronic kidney disease stages 1–5 and observed RDW was independently related to endothelial dysfunction (30). In addition, many studies revealed a closely association between RDW and disease activity in patients with IBD (31, 32). As mentioned above, these psoriasis associated conditions are all related to chronic inflammation and have a certain connection with RDW. Currently, the association between RDW and psoriasis was controversial and remained unclear among the representative and large sample of US population. Here, we detected the association between RDW and psoriasis risk in US adults and observed a positive association between RDW and psoriasis risk in females but not in males. RDW detection technology is mature and widely used as a testing indicator in clinical practice with the advantages of low cost, simplicity, and universality. Therefore, RDW could serve as promising predictive diagnostic biomarkers of psoriasis.

In patients with psoriasis, various biomarkers related to inflammation such as C-reactive protein, erythrocyte sedimentation rate, and platelet activation marker P-selectin are correlated with the severity of psoriasis (33–35). Naik and colleagues have provided evidence that the severity of psoriasis is linked to vascular inflammation detected through 18-fluorodeoxyglucose-positron emission tomography/computed tomography (FDG-PET/CT) (36), which indicated that psoriatic inflammation can affect blood vessels and leading to inflammation in the vascular wall. Inflammation is an important factor in the formation of erythrocyte anomalies, where abnormalities in the uptake and utilization of iron may impair erythropoiesis and lead to immature red blood cells entering the bloodstream (37). In addition, inflammation can decrease the survival rate of red blood cells, resulting in a higher value of RDW due to mixed numbers of red blood cells in circulation (38). Overall, these findings demonstrate the complex relationship between psoriatic inflammation, vascular inflammation, and erythrocyte anomalies. The results in our study may provide insights into the underlying mechanisms of psoriasis and aid in the development of effective treatments.

Previous study suggested a clear bimodal age pattern in psoriasis onset, and women tend to have a higher incidence of early (39). A questionnaire survey involving 1,979 psoriasis patients in Switzerland revealed that females experienced more severe pruritus symptoms compared to males (40). Our study found a positive association between RDW and psoriasis risk in females but not in males. We speculated that estrogen levels, and subcutaneous fat levels might play a physiological role in females somewhat, although it has not yet been discovered. In addition, subgroup analysis indicated that the association between RDW and psoriasis risk was more pronounced in female with BMI greater than 29.9 kg/m^2^. Previous study found that BMI was have a significant positive linear correlation with RDW and inflammatory index, such as WBC, neutrophil count, PCT and platelet count (41). A recent research observed a positive association between BMI and RDW in women (42). Given this, further research is needed to verify these results.

The strengths of this study include the use of a large and representative samples of the general adult population and adjustment for potential confounders. There are several limitations need to be acknowledged. Firstly, we can not detected the temporal relationship between RDW and psoriasis due to the nature of the cross-sectional design. Secondly, covariates relating to psoriasis and other medical history were evaluated using self-reported questionnaires, making our data susceptible to recall and information biases. Third, the RDW value can vary over time, and the dynamic value can not obtained from the database, which might interfere the analysis results. In addition, though we adjusted for various potential confounding variables associated with RDW and psoriasis, residual confounding is possible.

## Conclusion

In conclusion, our findings suggested that RDW was positively associated with psoriasis risk in adult females in United States. In addition, no association between RDW and psoriasis risk was found in males. The results may benefit patients by providing statistical data for clinicians in the early diagnosis of psoriasis. Future research using multi-center and prospective cohort study designs are needed to assess our results.

## Data availability statement

Publicly available datasets were analyzed in this study. This data can be found here: www.cdc.gov/nchs/nhanes.

## Ethics statement

The studies involving humans were approved by the board of the National Center for Health Statistics. The studies were conducted in accordance with the local legislation and institutional requirements. Written informed consent for participation in this study was provided by the participants’ legal guardians/next of kin.

## Author contributions

YZ: Conceptualization, Writing – original draft. ZL: Formal analysis, Writing – original draft. PP: Data curation, Formal analysis, Methodology, Writing – original draft. TZ: Conceptualization, Supervision, Writing – review & editing.
